# Beyond *KRAS* mutation status: influence of *KRAS* copy number status and microRNAs on clinical outcome to cetuximab in metastatic colorectal cancer patients

**DOI:** 10.1186/1471-2407-12-292

**Published:** 2012-07-17

**Authors:** Leonie JM Mekenkamp, Jolien Tol, Jeroen R Dijkstra, Inge de Krijger, M Elisa Vink-Börger, Shannon van Vliet, Steven Teerenstra, Eveline Kamping, Eugène Verwiel, Miriam Koopman, Gerrit A Meijer, J Han JM van Krieken, Roland Kuiper, Cornelis JA Punt, Iris D Nagtegaal

**Affiliations:** 1 Department of Medical Oncology, Radboud University Nijmegen Medical Centre, Nijmegen, The Netherlands; 2 Department of Pathology, Radboud University Nijmegen Medical Centre, PO Box 9101, 6500 HB, Nijmegen, The Netherlands; 3 Department of Epidemiology, Biostatistics and Health Technology Assessment, Radboud University Nijmegen Medical Centre, PO Box 9101, 6500 HB, Nijmegen, The Netherlands; 4 Department of Human Genetics, Radboud University Nijmegen Medical Centre, Nijmegen, The Netherlands; 5 Department of Medical Oncology, University Medical Center Utrecht, Utrecht, The Netherlands; 6 Department of Pathology, VU University Medical Center, Amsterdam, The Netherlands; 7 Department of Medical Oncology, Academic Medical Centre, University of Amsterdam, Amsterdam, The Netherlands

## Abstract

**Background:**

*KRAS* mutation is a negative predictive factor for treatment with anti-epidermal growth factor receptor (EGFR) antibodies in metastatic colorectal cancer (mCRC). Novel predictive markers are required to further improve the selection of patients for this treatment. We assessed the influence of modification of *KRAS* by gene copy number aberration (CNA) and microRNAs (miRNAs) in correlation to clinical outcome in mCRC patients treated with cetuximab in combination with chemotherapy and bevacizumab.

**Methods:**

Formalin-fixed paraffin-embedded primary tumour tissue was used from 34 mCRC patients in a phase III trial, who were selected based upon their good (n = 17) or poor (n = 17) progression-free survival (PFS) upon treatment with cetuximab in combination with capecitabine, oxaliplatin, and bevacizumab. Gene copy number at the *KRAS* locus was assessed using high resolution genome-wide array CGH and the expression levels of 17 miRNAs targeting *KRAS* were determined by real-time PCR.

**Results:**

Copy number loss of the *KRAS* locus was observed in the tumour of 5 patients who were all good responders including patients with a *KRAS* mutation. Copy number gains in two wild-type *KRAS* tumours were associated with a poor PFS. In *KRAS* mutated tumours increased miR-200b and decreased miR-143 expression were associated with a good PFS. In wild-type *KRAS* patients, miRNA expression did not correlate with PFS in a multivariate model.

**Conclusions:**

Our results indicate that the assessment of *KRAS* CNA and miRNAs targeting *KRAS* might further optimize the selection of mCRC eligible for anti-EGFR therapy.

## Background

Recent advances in our understanding of the specific signalling pathways of cancer cells have introduced targeted therapy into treatment regimes for patients with metastatic colorectal cancer (mCRC). Antibodies against the epidermal growth factor receptor (EGFR), cetuximab and panitumumab, have shown a survival benefit in mCRC patients with *KRAS* wild-type tumours both as monotherapy [1,2] and when added to chemotherapy[3,4]. Patients with a tumour harbouring a *KRAS* codon 12 or 13 mutation are resistant to anti-EGFR therapy[1,5]. Therefore the use of these antibodies is restricted to patients with *KRAS* wild-type tumours. However, within this subset not all patients respond to this treatment, and therefore additional predictive markers are needed. We have previously excluded a discordance in *KRAS* mutation status between the primary tumour and corresponding metastases as an explanation for the heterogeneous response rate in patients with *KRAS* wild-type tumours [6]. In routine practice, *KRAS* mutations in codons 12 and 13 are tested, which comprise approximately 96% of the observed *KRAS* mutations [7]. Recent data suggest that a codon 13 *KRAS* mutation has a distinct clinical behaviour and is not associated with cetuximab resistance [8]. Whether other *KRAS* mutations (like codon 61) result in similar resistance to EGFR monoclonal antibodies remains speculative [9]. A mutation in the *BRAF* oncogene occurs in approximately 10% of mCRC patients and is restricted to *KRAS* wild-type tumours, and was first shown to have a negative predictive value for anti-EGFR therapy [10]. Subsequently, we have shown that a *BRAF* mutation predominantly has a strong negative prognostic value [11]. Other biomarkers in the PI3K and RAS/MAPK pathways [12-16], ligands to the EGFR [17,18], and germline single nucleotide polymorphisms [19-21] have not yet shown a predictive value that can be used in clinical practice.

Point mutations in the *KRAS* oncogene lead to a significantly increased RAS-GTPase activity, ultimately resulting in the stimulation of cell proliferation and the inhibition of apoptosis via the RAS/MAPK pathway [22]. However, in addition to oncogenic mutations, copy number changes of the *KRAS* gene or posttranslational factors may also be involved in the regulatory mechanism of RAS-GTPase activity. Copy number aberrations (CNA) occur throughout the tumour genome and are an important mechanism in colorectal cancer development[23]. Genome-wide studies in mCRC patients have identified loci that are associated with a poor prognosis [24] and with the prediction of response to chemotherapy[25]. However, little is known about the prevalence and effect of CNA of the *KRAS* locus on chromosome 12p12.1. By using a TaqMan-based *KRAS* copy number assay, *KRAS* amplifications were observed in approximately 2% of the 106 investigated colorectal primary tumours [26]. In CRC cell lines, gains of the *KRAS* locus were shown to be associated with an eleven-fold increase in RAS-GTPase activity, which is comparable with the twelve-fold increase caused by a codon 12 or 13 mutation [27]. However, no data have been reported on CNA affecting the *KRAS* locus and their possible association with response to cetuximab.

In recent years, a rapidly expanding interest has manifested on microRNAs (miRNAs). These single stranded RNAs of 19–23 nucleotides regulate gene expression by translational inhibition or mRNA degradation via imperfect base pairing to the 3’-untranslated region (3’UTR) of their target mRNAs [28]. MiRNAs are involved in the development of human cancer, and in case of dysregulation they can act either as oncogenes or tumour suppressors, depending on their target genes [29]. Recently several miRNAs were identified that target *KRAS*, resulting in the suppression of cancer development [30-33]. *KRAS* contains multiple let-7 complementary sites, allowing the let-7 family of miRNAs to act as a tumour suppressor by regulating the *KRAS* mRNA [30,31]. Also mir-18a and miR-143 directly recognize *KRAS,* and downregulation of these miRNAs accelerates tumourigenesis by reversal of *KRAS* suppression [32]. The targeting effect of miR-18a on *KRAS* has been demonstrated in colon cancer cell lines irrespective of *KRAS* mutation status [33]. MiRNAs interfering with the RAS-signaling pathway may have predictive value or may even serve as targets for treatment. Currently no data are available on the clinical relevance of miRNAs involved in KRAS activity in patients treated with cetuximab.

In this study we analyzed the *KRAS* copy number status and the expression of miRNAs targeting *KRAS* in relation to clinical outcome in mCRC patients treated with first-line cetuximab-containing therapy.

## Methods

### Patients

The patients included in this study participated in the CAIRO2 trial (CKTO 2005–02; ClinTrials.gov NCT00208546) of the Dutch Colorectal Cancer Group (DCCG) [34]. In this multicenter phase III trial, 755 mCRC patients were randomized between first-line treatment with capecitabine (1000 mg/m2 bid.), oxaliplatin (130 mg/m2), and bevacizumab (7.5 mg/kg), or the same schedule with the addition of weekly cetuximab (250 mg/m2, after initial 400 mg/m2). Translational research on tumour tissue was part of the informed consent procedure. The primary end point of the study was progression free survival (PFS), and secondary end points were overall survival, response rate, and toxicity. The median PFS in patients treated with cetuximab was 9.4 months (95% CI 8.4-10.5 months), which was significantly shorter than the PFS of patients treated in the group without cetuximab (median PFS 10.7 months, 95% CI 9.7-12.3 months, p = 0.01). Patients in the cetuximab-group with a *KRAS* mutated tumour had a significantly decreased median PFS compared to patients with a *KRAS* wild-type tumour (8.1 versus 10.5 months, respectively, p = 0.04).

For the current analysis we selected patients who had been randomized to the cetuximab treatment arm, received at least three treatment cycles, did not discontinue treatment for other causes than disease progression, had a normal serum lactate dehydrogenase at randomisation, and of whom formalin-fixed paraffin-embedded (FFPE) material of the primary tumour as well as normal tissue was available. Patients with a rectal carcinoma and patients who had received preoperative radiotherapy on the pelvis have been excluded for these analyses. From this group the 17 best and 17 worst responding patients were selected based on both extremes of the PFS time. Throughout the article the terms good and poor responders are used, which does not apply to response according to RECIST, but to the patients with the longest and shortest PFS on cetuximab-based treatment. This outcome parameter was chosen for the current study because it is the best reflection of the clinical trial upon which this analysis is based. Next, especially with respect to targeted agents PFS appears to be superior of response rate in terms of clinical outcome.

### DNA extraction and mutation analysis

Genomic DNA was extracted from 4–8 manually micro dissected 50 μm sections of FFPE tissue as previously described [35]. DNA concentration was determined using the Nanodrop ND-1000 spectrophotometer (Nanodrop Technologies Inc., Wilmington, USA). DNA quality was assessed by performing a multiplex PCR using 4 primer sets, resulting in fragments of 100, 200, 300 and 400 base pairs [36]. The *KRAS* mutation status [35] and *BRAF* mutation status [11,12] were assessed by sequencing analysis as previously described.

### Assessment of the *KRAS* gene copy number and data analysis

High-resolution genome-wide DNA copy number profiles were generated by array-based comparative genomic hybridization (array CGH) using 720 k Whole-Genome Tiling CGH arrays (Roche NimbleGen Inc., Madison, USA). Optimal signal-to-noise ratios were obtained by hybridizing test (tumour) and reference (normal colon) DNA of similar quality, which was determined by giving similar yield in a Bioscore Screening and Amplification kit (ENZO diagnostics Inc., Farmingdale, USA). For hybridization, 500 ng of amplified DNA from test and reference samples were labelled with Cy3 and Cy5, respectively, using random-primed labelling (Bioprime genomic DNA labelling kit, Invitrogen, Breda, the Netherlands), and hybridized for 48 hours at 42°C using a MAUI hybridization system (Biomicro Systems, Salt Lake City, USA). After washing, arrays were scanned in an Axon Genepix 4200AL microarray scanner. The NimbleScan 2.4 software package (NimbleGen Systems Inc., Madison, USA) was used to calculate log2 ratios after performing spatial correction, normalization and a 25 kb average smoothing window on the data. Further data interpretation and CNA calling was done with Nexus Copy Number 5.0 software (Biodiscovery, El Segundo, USA) using the Rank Segmentation Algorithm. In 26 patients, hybridizations were performed against normal DNA from the same patient to normalize for germline copy number changes. In the other 8 cases, germline copy number changes were excluded using both public (http://projects.tcag.ca/variation/) and private CNA databases. The cut-off value for gene copy number gain and loss were manually set for each sample to adjust for differences in signal strength and incorrectly centered baselines.

### Prevalence of *KRAS* locus gene copy number changes

In order to assess the clinical relevance in terms of prevalence of *KRAS* gene copy number changes in mCRC patients, we assessed the *KRAS* gene copy number status in FFPE primary tumour tissue of 225 unselected mCRC patients who participated to our previous phase III study which did not involve the use of targeted agents [37]. In these patients a 250 k oligonucleotide array CGH was performed as previously described[25].

### Multiplex Ligation-dependent Probe Amplification (MLPA)

MLPA was performed according to the manufacturer’s instruction using the SALSA P145-A2 kit (MRC Holland, Amsterdam, the Netherlands), containing 40 probes including the 12p12.1 *KRAS* probe. Briefly, 200 ng DNA was denatured and allowed to hybridize for 16 h at 60°C in a thermocycler. Then SALSA Ligase-65 enzyme was added and ligation was allowed at 54°C. After heat inactivation of the ligase enzyme at 98°C, primers, dNTPs and polymerase were added and PCR amplification was performed for 35 cycles (60s at 95°C, 30s at 60°C, and 90s at 72°C). Reactions were performed on a PTC 200 thermal cycler (MJ Research Inc., Waltham, Massachusetts, USA). One microliter of PCR product was analysed by capillary electrophoresis on an ABI 3730 Analyzer (Applied Biosystems), and quantitative data were obtained by Genemapper analysis (Applied Biosystems).

### MLPA data analysis

For each tumour sample, the peak area of the 12p12.1 and reference probes were determined in duplicate for further analysis. The reference peak area was obtained from blood samples from three different individuals, each of which were analysed at least two times independently. In every sample, for every probe, a tumour to normal DNA copy number ratio was calculated by dividing the median area under the peak for the 12p12.1 probe by the value for the reference DNA. Subsequently, all ratios were normalized by setting the median tumour to normal DNA copy number ratio of the reference genes in de probe mixture to 1.0. A ratio lower than 0.8 was considered a loss and a ratio higher than 1.2 a gain.

### MiRNA selection

Selection of miRNAs regulating *KRAS* was performed using PicTar (http://pictar.mdc-berlin.de/), TargetScan (http://www.targetscan.org/) and miRNA targets (http://cbio.mskcc.org/mirnaviewer/). Venn diagram analysis was used to select 14 miRNAs who were identified by at least two algorithms (Additional file 1: Figure S1). In addition to the prediction programs we also selected six extra miRNAs (Let-7, miR-18a, miR-21, miR-133a, miR-133b, miR-205) which have been shown to target *KRAS* in previous studies [30,33,38], resulting in a total test series of 20 miRNAs. Two Taqman microRNA assay were not available (mir-18a, mir-200c), resulting in 18 miRNAs to analyze.

### Total RNA extraction, miRNA reverse transcription and real-time PCR

 Total RNA was isolated from FFPE tissue of 34 primary tumours and matched normal tissue using the RecoverAll™ Total Nucleic Acid Isolation Kit (Applied Biosystems, Foster city, USA). In brief, four tissue slices of 20 μm were micro dissected and incubated with 100% xylene at 50°C to remove paraffin excess, followed by ethanol washes. Proteins were degraded by protease at 50° and 80°C. The RNA was extracted followed by nuclease digestion. Total RNA quantity and quality were determined using the Nanodrop 26 ND-1000 spectrophotometer (Nanodrop Technologies Inc., Wilmington, USA).

To determine the expression levels of miRNAs*,* Taqman microRNA assays directed to seventeen miRNAs and the endogenous reference gene (RNU 6B) were used following the manufacture’s protocol (Applied Biosystems, Foster City, USA). Firstly, cDNA was synthesized in duplicate from total RNA using miRNA specific stem loop primers. Reverse transcriptase reactions were conducted using 10 ng total RNA, 1 mM dNTPs, 50 U MultiScribe^TM^ Reverse Transcriptase, 1 x RT buffer, 3.8 U RNase inhibitor and 1 x Taqman® MicroRNA RT Primer (Applied Biosystems, Foster city, USA). The 15 μl reactions were incubated at 16°C for 30 minutes at 42°C for 30 minutes and at 85°C for 5 minutes.

Secondly, the quantitative PCR was performed in which the total mixture of 20 μl included 1.33 μl RT product (1:5 diluted from RT reaction), 1 x Taqman® Universal PCR Master Mix (No AmpErase® UNG, Applied Biosystems, Foster City, USA) and 1 x the dedicated primer and probe mix. The reactions were incubated in a 96-well optical plate at 95°C for 10 minutes, followed by 40 cycles at 95°C for 15 seconds and at 60°C for 1 minute. All reactions were carried out in duplicate in a 7500 Real Time PCR System (Applied Biosystems, Foster City, USA). The threshold cycle (Ct) was defined as the fractional cycle number at which the fluorescence passes the fixed threshold. Relative quantification of miRNA expression was calculated using the ΔΔCt method as described previously [39].

### Statistical analysis

PFS was defined as the interval from the date of randomization to the date of first documented disease progression or death, whichever occurred first. Statistical differences of clinical and pathological parameters between good and poor responders were evaluated using the Student’s t-test, Pearson’s χ^2^ test or Fisher’s exact test where appropriate. The miRNA expression in colorectal tumours was described by the relative quantity (RQ) of the target miRNA, normalized in respect to RNU6B and relative to matched normal tissue. Box plots were used to appreciate the descriptive statistics of the data. Differences in expression of the target miRNA between good and poor responders were evaluated on the log scale (ΔΔCt scale) to obtain normally distributed data. The Student’s t-tests was used in exploratory analyses on the miRNA expression in relation to response and to *KRAS* mutation status. When focusing on the actually observed PFS, we investigated by Cox regression analysis the influence of each miRNA on PFS, using *KRAS* mutation status, the interaction term between miRNA and *KRAS* mutation, and differentiation grade as covariates. Due to the limited number of patients and the ensuing risk for overfitting, it was not possible to assess the influence of all miRNAs together (i.e. correct the influence of miRNA for each other), nor to correct for other baseline characteristics.

## Results

### Patients

Of the 34 patients selected for this analysis, the median PFS was 22.5 months (range 14.8-39.8 months) in the 17 good responders, and 6.0 months (range 2.3-7.2 months) in the 17 poor responders. Clinical and pathological characteristics of the primary tumour were well balanced between the 17 good and 17 poor responders. Only poor differentiation grade of the primary tumour was more frequently observed in the poor responders.

A *KRAS* mutation was demonstrated in the primary tumour of 15 patients (6 good responders and 9 poor responders), and *KRAS* wild-type in the primary tumour of 19 patients (11 good responders and 8 poor responders). *KRAS* codon 12 mutation was observed in 14 patients, and one poor responder had a codon 13 mutation. Of the *KRAS* wild-type patients, 4 had a *BRAF* mutated tumour (1 good responder and 3 poor responders) (Table 1).

**Table 1:** Clinical and histopathological characteristics of patients and their respective tumours

		**All eligible patients n = 34**	**Good responders n =17**	**Poor responders n =17**	**p-value**
**Age**	Mean	58.6	58.0	59.2	0.07
**Gender**	Female	14 (41%)	5 (29%)	9 (53%)	0.30
	Male	20 (59%)	12 (71%)	8 (47%)	
**Number of metastatic sites**	1	17 (50%)	9 (53%)	8 (47%)	0.60
	>1	17 (50%)	8 (47%)	9 (53%)	
**WHO PS**	0	23 (68%)	11 (65%)	12 (71%)	0.71
	1	11 (32%)	6 (35%)	5 (29%)	
**Site of primary tumour**	Colon	22 (65%)	11 (65%)	11 (65%)	0.90
	Rectosigmoid	12 (35%)	6 (35%)	6 (35%)	
**T stage**	1-2	4 (12%)	2 (12%)	2 (12%)	0.49
	3	21 (62%)	12 (71%)	9 (53%)	
	4	9 (26%)	3 (18%)	6 (35%)	
**N stage**	0	9 (26%)	4 (24%)	5 (29%)	0.61
	1	8 (24%)	4 (24%)	4 (24%)	
	2	14 (41%)	9 (53%)	5 (29%)	
	Unknown	3 (9%)	0	3 (18%)	
**Differentiation grade**	Good	1 (3%)	1 (6%)	0	0.02
	Moderate	23 (68%)	15 (88%)	8 (47%)	
	Poor	10 (29%)	1 (6%)	9 (53%)	
	Wild-type	30 (88%)	16 (94%)	14 (82%)	0.29
	Mutant	4 (12%)	1 (6%)	3 (18%)	
***KRAS*mutation status**	Wild-type	19 (56%)	11 (65%)	8 (47%)	0.30
	Mutant	15 (44%)	6 (35%)	9 (53%)	
***KRAS*mutation type**	Codon 12	14 (93%)	6 (100%)	8 (89%)	0.40
	Codon 13	1 (7%)	0	1 (11%)	
**PFS (months)**	Median (range)	11.0 (2.3-39.8)	22.5 (14.8-39.8)	6.0 (2.3-7.2)	s<0.0001

Abbreviations: PS = performance status, PFS = progression-free survival.

### 12p12.1 copy number changes in good and poor responders

By using high resolution array CGH, two copy number gains (of which one amplification) and 5 losses were detected at the 12p12.1 locus where *KRAS* is localized (Additional file 2: Figure S2). Both copy number gains, which were confirmed by MLPA, were observed in poor responders with a *KRAS* wild-type tumour. Of these tumours one sample contained a gain of the complete p-arm of chromosome 12 and the other sample contained a high copy number gain of a region including the *KRAS* locus.

**Figure 1:**
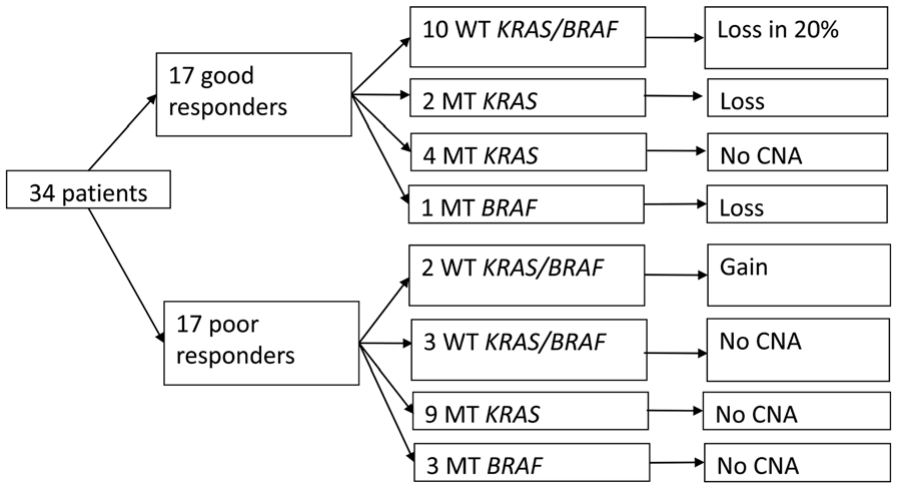
**12p12.1 copy number changes in good and poor responders according to the*KRAS*mutation status.** Abbreviations: CNA = copy number aberration. MT = mutant,Â WTÂ = wild-type.

A 12p12.1 copy number loss, detected by array CGH, was observed in the tumour of 5 patients with a good response. One tumour contained a loss of the whole chromosome, three tumours included a loss of the short arm of the chromosome and one tumour contained a loss of a 27.5 Mb region of the short arm of chromosome 12 including the *KRAS* locus (Additional file 2: Figure S2). Of these 5 tumours with loss of the 12p12.1 locus, 2 tumours harboured a *KRAS* mutation, and one tumour had a *BRAF* mutation, suggesting that the mechanism of gene copy number loss is independent of the *KRAS* and *BRAF* mutation status (Figure 1).

### 12p12.1 gene copy number changes in a control group of mCRC patients

In an unselected group of 222 mCRC patients from our previous trial with comparable baseline characteristics[37], the prevalence of 12p12.1 copy number changes was assessed. In this group three amplifications (1.4%), 32 copy number gains (14.4%), and 12 losses (5.4%) of the 12p12.1 locus were observed. There was no effect of *KRAS* copy number gain or loss on prognosis in these patients treated with first-line chemotherapy without cetuximab (p = 0.97 and p = 0.75, respectively, data not shown).

### MiRNA expression in good and poor responders

To assess the role of miRNA expression in relation to clinical outcome, the expression levels of 18 miRNAs targeting *KRAS* were determined by real-time RT-PCR in 32 primary colorectal tumours relative to their matched normal tissue. Two patients (1 good and 1 poor responder) were not accessible for miRNA expression due to an insufficient RNA amount in normal mucosa. MiR-205 expression was undetectable in both tumour and normal mucosa, therefore 17 miRNAs were included in our final analysis. By using NormFinder [40] and GeNorm [41], the use of RNU6B as a reference gene was justified.

The expression level of 14 miRNAs showed a trend towards a higher expression in patients with a good response compared to patients with a poor response, however this trend was not statistically significant. MiR-143, miR-133a and miR-133b expression was decreased in patients with a good response, of which miR-143 showed a relative expression in good versus poor responders of 0.49 (p = 0.07) (Table 2).

**Table 2:** MiRNA expression in good versus poor responders

	**Good responders**			**Poor responders**			**RQ Good versus poor**	**P value**
	**Mean ddCt**	**SE**	**RQ**	**Mean ddCt**	**SE**	**RQ**		
**MiR-27b**	−0.98	0.77	1.98	−0.92	0.64	1.90	1.04	0.80
**MiR-105**	−1.04	2.80	2.05	0.57	3.15	0.68	3.01	0.21
**MiR-155**	−0.66	1.25	1.58	−0.19	0.89	1.14	1.39	0.24
**MiR-346**	0.32	1.52	0.80	0.57	1.14	0.67	1.19	0.60
**MiR-181a**	−1.64	0.85	3.11	−1.27	0.75	2.42	1.29	0.21
**MiR-19a**	−3.39	1.84	10.45	−2.82	1.55	7.04	1.48	0.35
**MiR-200b**	−0.57	0.92	1.49	0.05	1.42	0.97	1.54	0.15
**MiR-27a**	−2.10	0.98	4.27	−2.00	0.82	4.00	1.07	0.77
**MiR-30a**	−0.81	0.86	1.75	−0.66	0.88	1.58	1.11	0.65
**Let-7a**	−0.67	0.79	1.59	−0.39	0.70	1.31	1.21	0.30
**MiR-21**	−3.16	1.16	8.92	−2.75	0.79	6.75	1.32	0.26
**MiR-96**	−4.56	1.32	23.59	−3.81	1.40	14.04	1.68	0.13
**MiR-143**	−0.73	1.35	1.66	−1.76	1.55	3.38	0.49	0.07
**MiR-217**	−2.91	3.09	7.53	−1.64	3.10	3.12	2.41	0.30
**MiR-133a**	0.91	1.69	0.53	0.33	1.79	0.79	0.67	0.36
**MiR-133b**	0.98	1.74	0.51	0.39	2.23	0.76	0.67	0.41
**MiR-19b**	−2.95	1.71	7.73	−2.45	1.37	5.45	1.42	0.37

Abbreviations: SE = standard error, RQ = relative quotient.

### MiRNA expression in good and poor responders according to *KRAS* mutation status

 In patients with a wild-type *KRAS* tumour, the expression level of miR-181a showed a 1.87-fold increase in good responders compared to poor responders (p = 0.04), which was not observed in patients with mutated *KRAS* tumours (0.91-fold increase, p = 0.69). A higher expression of miRNAs in wild-type *KRAS* good responders compared with wild-type *KRAS* poor responders was also observed for MiR-200b (2.48-fold increase, p = 0.01) and miR-21 (1.66-fold increase, p = 0.06).

**Figure 2:**
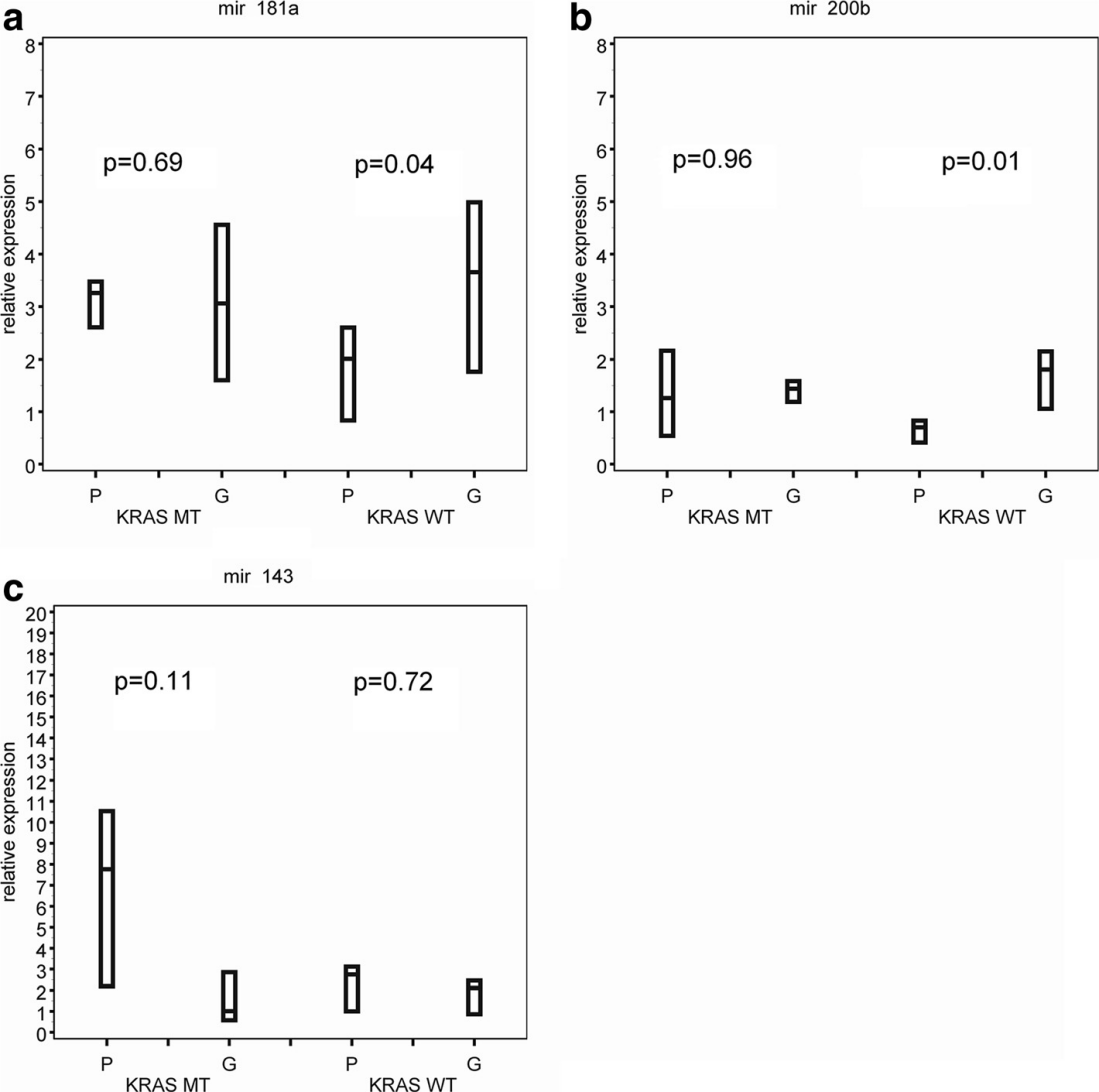
**Box plots of the expression levels of miR-181a, miR-200b and miR-143 in mCRC patients according to clinical outcome and*KRAS*mutation status.** Abbreviations: G = good responders, P = poor responders, MT = mutant, WT = wild-type.

A difference between the expression of miR-143 in good versus poor responders was more obvious in mutated *KRAS* tumours. The relative expression level of miR-143 showed a 0.30 fold increase in mutated *KRAS* good responders versus mutated *KRAS* poor responders (p = 0.11) (Figure 2).

### Multivariate model of PFS in relation to miRNA expression and *KRAS* mutation status

Each miRNA was analyzed individually together with differentiation grade as a covariate for PFS in wild-type *KRAS* and mutated *KRAS* patients treated with first-line cetuximab-containing therapy (Table 3). Differentiation grade was used as a covariate in the Cox regression model because this pathological feature is a well known prognostic factor and differentially distributed between good and poor responders.

**Table 3:** **A multivariate model in which each miRNA was analyzed individually together with differentiation grade as a predictor for PFS in wild-type-*KRAS*and mutated-*KRAS*patients treated with chemotherapy, bevacizumab and cetuximab**

		**Overall n = 32**	***KRAS *wild-type n = 18**	***KRAS *mutation n = 14**	**p-value**
**MiR-27b**	HR (95% CI)	0.74 (0.38-1.42)	0.94 (0.36-2.42)	0.51 (0.20-1.32)	0.38
	p-value	0.36	0.89	0.16	
**MiR-105**	HR (95% CI)	1.04 (0.84-1.29)	0.95 (0.76-1.21)	1.22 (0.83-1.78)	0.26
	p-value	0.72	0.69	0.32	
**MiR-155**	HR (95% CI)	0.92 (0.62-1.35)	0.77 (0.38-1.53)	0.96 (0.60-1.51)	0.60
	p-value	0.66	0.45	0.85	
**MiR-346**	HR (95% CI)	0.83 (0.63-1.10)	0.85 (0.61-1.19)	0.72 (0.40-1.32)	0.63
	p-value	0.20	0.34	0.29	
**MiR-181a**	HR (95% CI)	0.75 (0.46-1.22)	0.70 (0.37-1.33)	0.94 (0.44-2.02)	0.57
	p-value	0.24	0.27	0.87	
**MiR-19a**	HR (95% CI)	0.96 (0.78-1.19)	1.03 (0.80-1.33)	0.74 (0.45-1.20)	0.23
	p-value	0.73	0.82	0.22	
**MiR-200b**	HR (95% CI)	1.03 (0.72-1.47)	0.90 (0.51-1.57)	1.78 (0.87-3.62)	0.18
	p-value	0.86	0.70	0.10	
**MiR-27a**	HR (95% CI)	0.74 (0.43-1.27)	0.69 (0.31-1.54)	0.71 (0.35-1.43)	0.96
	p-value	0.27	0.37	0.34	
**MiR-30a**	HR (95% CI)	0.70 (0.44-1.10)	0.77 (0.40-1.55)	0.70 (0.37-1.34)	0.86
	p-value	0.12	0.46	0.28	
**Let-7a**	HR (95% CI)	0.94 (0.59-1.50)	0.89 (0.50-1.59)	1.28 (0.46-3.58)	0.55
	p-value	0.80	0.68	0.63	
**MiR-21**	HR (95% CI)	0.89 (0.57-1.39)	1.15 (0.52-2.55)	0.81 (0.49-1.33)	0.47
	p-value	0.61	0.74	0.40	
**MiR-96**	HR (95% CI)	0.84 (0.59-1.20)	0.74 (0.45-1.22)	0.93 (0.55-1.58)	0.54
	p-value	0.33	0.24	0.79	
**MiR-143**	HR (95% CI)	0.73 (0.51-1.05)	0.92 (0.56-1.51)	0.63 (0.40-0.99)	0.26
	p-value	0.09	0.74	0.04	
**MiR-217**	HR (95% CI)	1.01 (0.87-1.16)	0.85 (0.61-1.19)	0.78 (0.56-1.08)	0.72
	p-value	0.95	0.34	0.13	
**MiR-133a**	HR (95% CI)	0.81 (0.63-1.05)	0.98 (0.68-1.43)	0.66 (0.41-1.06)	0.21
	p-value	0.12	0.92	0.09	
**MiR-133b**	HR (95% CI)	0.89 (0.70-1.13)	1.04 (0.73-1.49)	0.80 (0.55-1.17)	0.32
	p-value	0.32	0.83	0.25	
**MiR-19b**	HR (95% CI)	0.95 (0.75-1.20)	1.02 (0.77-1.34)	0.66 (0.36-1.23)	0.21
	p-value	0.65	0.91	0.19	

Elevated expression of mir-200b was associated with a better PFS in patients with a mutated *KRAS* tumour (HR 0.56 (0.28-1.15); p = 0.10). This trend was not present in patients with a wild-type *KRAS* tumour. Surprisingly, increased expression of miR-143 resulted in a shorter PFS in patients with a mutated *KRAS* tumour (HR 1.59 (1.01-2.50); p = 0.04). The hazard ratio for PFS was not influenced by miR-143 expression in wild-type *KRAS* tumours.

## Discussion

We demonstrated that regulation of the *KRAS* oncogene at several levels might affect clinical outcome in a selected group of cetuximab-treated mCRC patients treated in a phase III trial [34]. Copy number loss of the *KRAS* locus was restricted to good responders, whereas a copy number gain was associated with a poor PFS in patients with wild-type *KRAS* tumours. Increased expression of miR-200b that targets *KRAS* was associated with improved PFS in patients with a mutated *KRAS* tumour. Surprisingly, decreased miR-143 expression was correlated with improved PFS in these patients.

The predictive strength of *KRAS* mutation status stresses the importance of RAS-GTPase activity for the response to cetuximab. Therefore, other regulatory mechanisms of RAS-GTPase activity are obvious novel candidate markers. CNA of the *KRAS* locus occur independently of the *KRAS* mutation status in a considerable percentage of colorectal tumours (21.2%) as assessed in a large and unselected mCRC population. Previously, it has been shown that *KRAS* copy number gains are correlated with increased RAS-GTPase activity in colorectal cell lines and with worse clinical outcome in lung adenocarcinomas [27]. Our results suggest that *KRA*S copy number gains are associated with worse clinical outcome in wild-type *KRAS* mCRC patients who are treated with a cetuximab-containing first-line regimen. This influence of *KRAS* copy number gain on prognosis was absent in mCRC patients treated without cetuximab, suggesting a predictive effect on cetuximab response. The correlation between miRNAs targeting *KRAS* and PFS was absent in wild-type *KRAS* patients. Inhibition of *KRAS* translation by miRNAs is probably only relevant when the *KRAS* expression levels are high, which is not the case in absence of an activating *KRAS* mutation.

*KRAS* mutations occur in approximately 38% of mCRC patients [7], and these patients are currently excluded from treatment with anti-EGFR antibodies. However, in our selected good responders, 6 patients (35%) had a *KRAS* mutated tumour. A recent publication showed that patients with codon 13-mutated tumours might benefit from cetuximab treatment [8]. In the current series none of the good responders had a tumour with a *KRAS* codon 13 mutation. Our data show that the presence of *KRAS* copy number loss in two of the mutated *KRAS* mCRC patients might justify treatment with cetuximab. Decreased expression of *KRAS* caused by loss of gene copies in correlation with response to cetuximab has not been described earlier. Despite the limitations in sample size and concomitant treatment our results indicate that patients with *KRAS* copy number loss might benefit from treatment with an anti-EGFR antibody although their tumour is *KRAS* mutated.

Next, we demonstrated that increased expression of miR-200b was associated with improved PFS in mutated *KRAS* patients. We hypothesize that reducing KRAS protein levels in the presence of a mutation might improve clinical outcome in patients treated with cetuximab. *KRAS* is not the only target of the miR-200 family, miR-200b is also capable of reducing *ERRFI-1* mRNA and subsequent activation of EGFR [42]. Adam et al. showed that increased expression of miR-200b facilitates optimal EGFR functionality, resulting in an efficient response of bladder cancer cells to cetuximab. To our knowledge, our results are the first data *in vivo* suggesting that in the presence of a *KRAS* mutation, an increased miR-200b expression is associated with an improved PFS in cetuximab-treated mCRC patients. Surprisingly a decreased expression of miR-143 was associated with improved PFS in patients with mutated *KRAS* tumours. MiR-143 is thought to inhibit *KRAS* translation and thereby to suppress tumour cell growth during tumourigenesis [32]. In an established tumour, modulation of *KRAS* by miR-143 may be differentially regulated which could possibly explain our findings. However, since miRNAs are capable of repressing over a hundred different mRNAs [28], miR-143 could also target mRNAs which may be relevant in response to capecitabine, oxaliplatin and bevacizumab. Previous studies on biomarkers have shown divergent results which stresses the importance of the results of our current hypothesis-generating study being confirmed in a larger, independent series of mCRC patients preferably treated with cetuximab monotherapy.

The patients used in this study were derived from a clinical trial, and the observed outcome is also influenced by the effect of the other agents used. The relative contribution of cetuximab to this outcome is therefore unclear. The phase III CAIRO2 trial showed that cetuximab plus chemotherapy and bevacizumab resulted in a significantly decreased median PFS compared to treatment with chemotherapy and bevacizumab alone. The explanation of this detrimental outcome is unclear [43], and complicates the interpretation of the current analysis. Excessive toxicity in the cetuximab group does not appear to be cause of these results. Negative interaction between the antibodies or between antibodies and chemotherapy might have influenced the outcome although preclinical observations supporting this hypothesis are not yet available. The interpretation of the current analysis is complicated by the detrimental outcome of the trial. Whether this outcome also affects the PFS in the good responders remains unclear.

In conclusion, the analysis of *KRAS* CNA and miRNAs targeting *KRAS* may optimize the selection of mCRC patients eligible for anti-EGFR therapy. Elevated expression of miR-200b, decreased miR-143 level and copy number losses may identify patients with mutated *KRAS* tumours who benefit from anti-EGFR therapy, whereas copy number gains in wild-type *KRAS* patients could predict resistance to cetuximab. Our results are relevant for the development of predictive biomarkers for anti-EGFR therapy, and suggest that the clinical effects of KRAS are the result of a complex interaction of several regulatory mechanisms beyond the *KRAS* point mutation status.

## Conclusions

KRAS activity, an important regulator of response to anti-EGFR therapy, can be influenced by genetic and epigenetic regulation. CNA and specific miRNAs may provide important additional information to *KRAS* mutation status and their use could further improve the selection of mCRC patients for anti-EGFR therapy. The hypothesis-generating nature of our study urges for our results to be confirmed in larger series.

## Competing interests

The authors declare that they have no competing interests.

## Authors’ contributions

LJMM, JT, JRD, IdK, MEVB, SvV, EK, and EV carried out the experiments. LJMM, JT, JRD, EV, ST, RK and IDN analysed and interpreted data. LJMM and JT were responsible for the study design and wrote the paper. MK, GAM, JHJMvK, RK, CJAP and IDN revised the manuscript critically. All authors were involved in editing the paper and had final approval of the submitted and published versions.

## Pre-publication history

The pre-publication history for this paper can be accessed here:

http://www.biomedcentral.com/1471-2407/12/292/prepub

## Supplementary Material

Additional file 1 **Figure S1.** Selection of MiRNAs that were identified by multiple algorithms. Click here for file

Additional file 2 **Figure S2.** Heat map representation and individual array CGH plots of patients with *KRAS* copy number aberrations. **A:** Heat map representation of the 7 patients with CNA of the *KRAS* locus. Each row represents a patient with a CNA of the *KRAS* locus (loss, gain, amplification). Whole chromosome 12, containing the *KRAS* locus, is depicted on the horizontal axis. **B:** Amplification of a genomic region in 12p12.1 detected by array CGH in two patients, as confirmed by MLPA. The DNA log2 ratios and whole chromosome 12 are represented on the vertical and horizontal axis, respectively. **C:** Array CGH plot of chromosome 12 of a patient with a deletion of *KRAS*, which could not be validated using MLPA, but was detected by the Nexus copy number algorithm. Most of the genomic deletions detected by array CGH appeared to be present subclonal, below the detection threshold of MLPA. The DNA log2 ratios and whole chromosome 12 are represented on the vertical and horizontal axis, respectively. Abbreviations: ampl = amplification. (TIFF 12705 kb) Click here for file
