# Supplementary figures and images for: Beyond KRAS mutation status: influence of KRAS copy number status and microRNAs on clinical outcome to cetuximab in metastatic colorectal cancer patients
Source: BMC Cancer. 2012 Jul 17;12:292. doi: 10.1186/1471-2407-12-292 (PMC3508829; doi:10.1186/1471-2407-12-292)

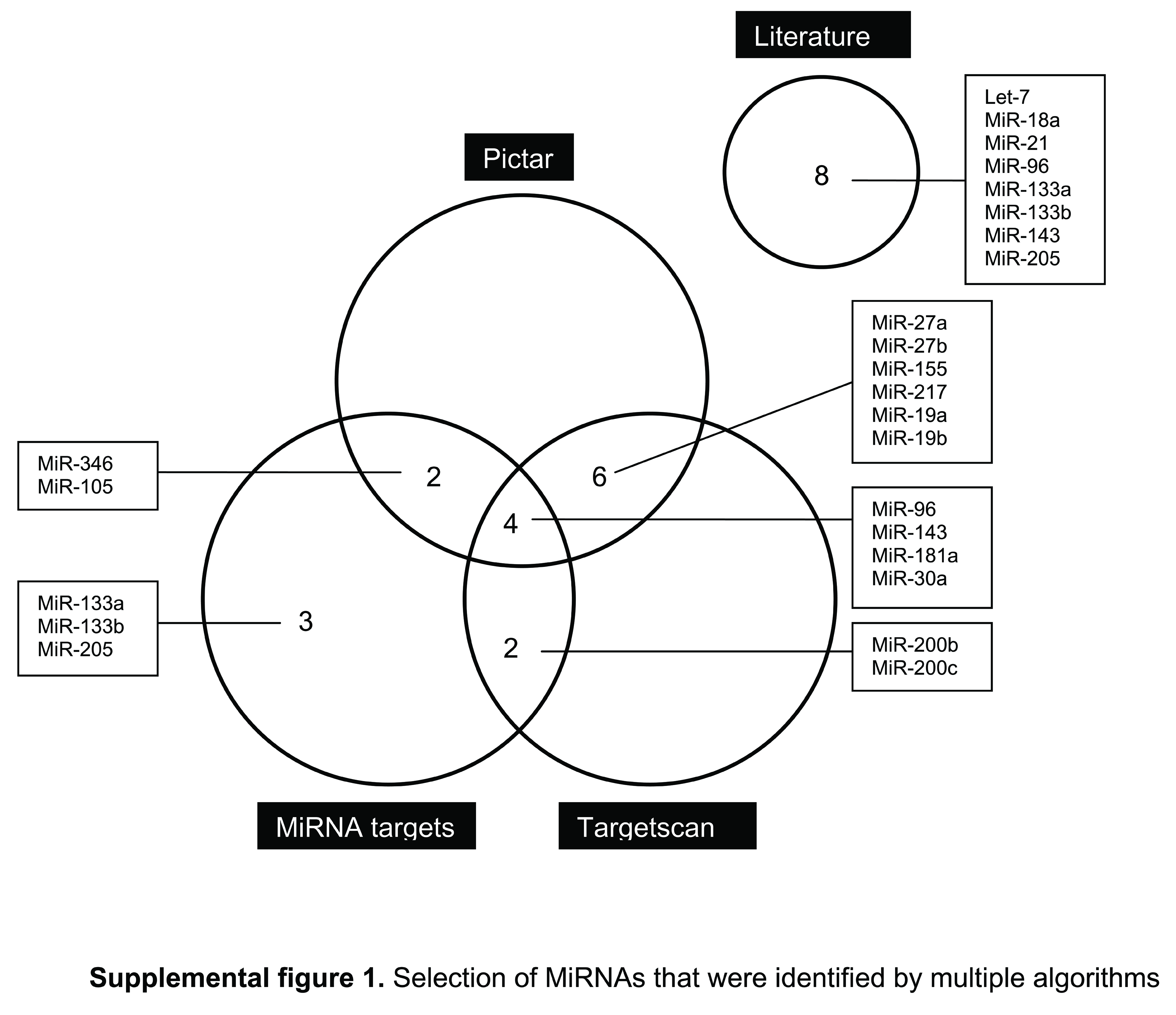

Supplement: Additional file 1 — Figure S1. Selection of MiRNAs that were identified by multiple algorithms. [file 1471-2407-12-292-S1.tiff]

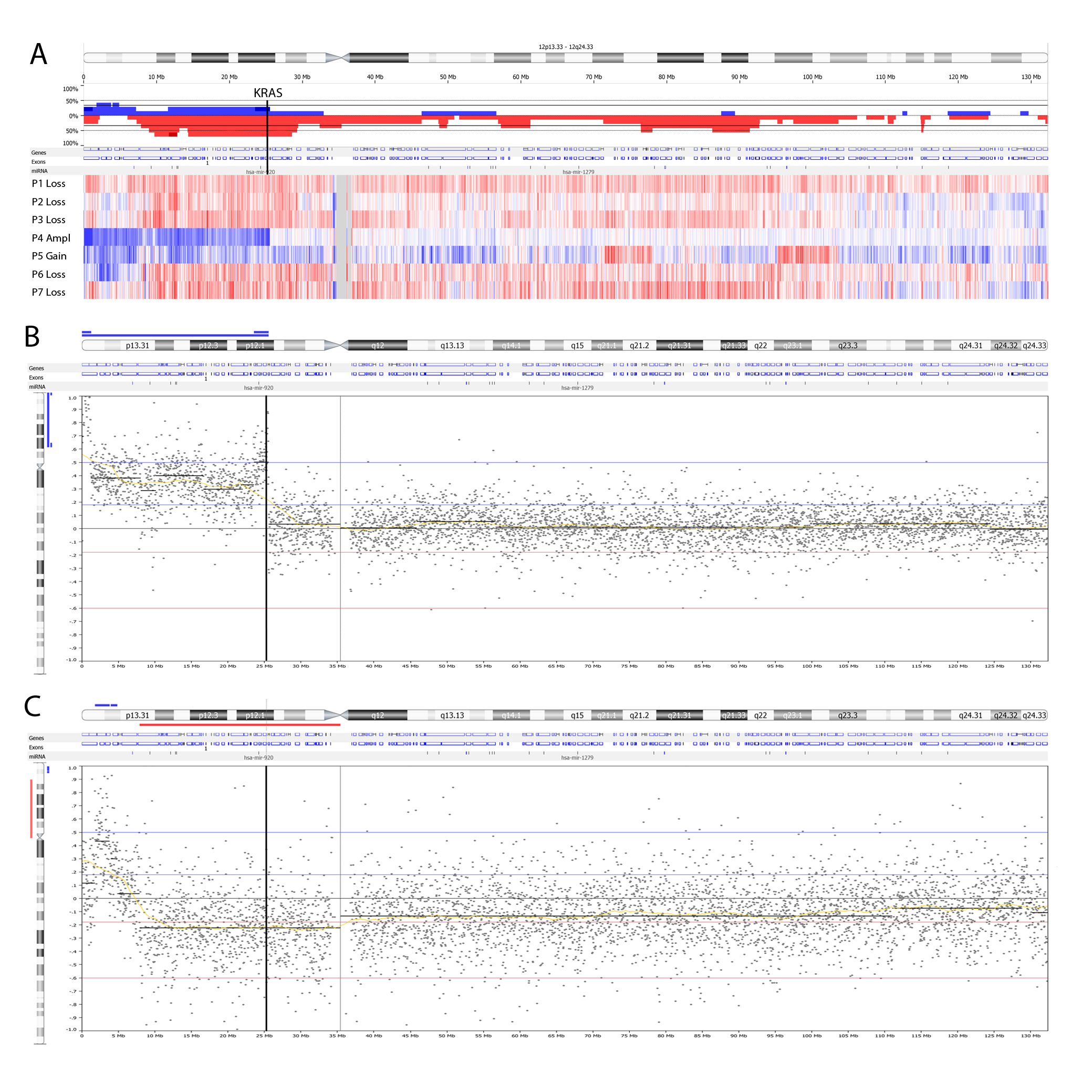

Supplement: Additional file 2 — Figure S2. Heat map representation and individual array CGH plots of patients with KRAS copy number aberrations. A: Heat map representation of the 7 patients with CNA of the KRAS locus. Each row represents a patient with a CNA of the KRAS locus (loss, gain, amplification). Whole chromosome 12, containing the KRAS locus, is depicted on the horizontal axis. B: Amplification of a genomic region in 12p12.1 detected by array CGH in two patients, as confirmed by MLPA. The DNA log2 ratios and whole chromosome 12 are represented on the vertical and horizontal axis, respectively. C: Array CGH plot of chromosome 12 of a patient with a deletion of KRAS, which could not be validated using MLPA, but was detected by the Nexus copy number algorithm. Most of the genomic deletions detected by array CGH appeared to be present subclonal, below the detection threshold of MLPA. The DNA log2 ratios and whole chromosome 12 are represented on the vertical and horizontal axis, respectively. Abbreviations: ampl = amplification. (TIFF 12705 kb) [file 1471-2407-12-292-S2.tiff]
